# The effects of Tween-80 on the integrity of solutions of capsaicin: useful information for performing tussigenic challenges

**DOI:** 10.1186/1745-9974-4-3

**Published:** 2008-05-27

**Authors:** Scott E Kopec, Richard S Irwin, Ronald J DeBellis, Mark B Bohlke, Timothy J Maher

**Affiliations:** 1 The Division of Pulmonary, Allergy, and Critical Care Medicine, UMASS Memorial Medical Center, Worcester, MA, USA; 2 Massachusetts College of Pharmacy and Health Sciences, Boston, MA, USA

## Abstract

**Background:**

Because variable results of capsaicin challenges may be due to the incomplete solubility of capsaicin, we sought to determine if the use of Tween-80 in solutions of capsaicin improves actual concentrations of freshly prepared and stored solutions.

**Methods:**

Capsaicin solutions ranging from 0.5–128 μM were mixed with and without Tween-80. Samples of various concentrations were then stored under 4 environmental conditions: 4°C, protected from light; room temperature, protected from light; room temperature, exposed to light; -20°C. All samples were analyzed initially, and at 2 and 4 months.

**Results:**

While freshly prepared solutions with Tween-80 had consistently higher concentrations than those prepared without Tween-80 (83% vs. 69%), Tween-80 does not facilitate complete solubility. For solutions stored at 4°C and protected from light, there was a significant decrease after 2 months in low concentration solutions of both the Tween-80 and non-Tween-80 solutions. Both Tween-80 and non-Tween-80 containing solutions significantly decreased in concentration after 2 months when stored at room temperature and protected from light, room temperature and exposed to light, and -20°C. Concentrations of solutions made of 4 μM or higher are stable when stored at 4°C and protected from light for 4 months.

**Conclusion:**

While the inherent difficulty of forcing capsaicin into solution cannot be eliminated, it can be improved with Tween-80. However, the addition of Tween-80 does not prevent the breakdown of stored capsaicin solutions. We recommend preparing and storing capsaicin solutions according to the methods and results of this study.

## Backround

Inhalational challenges using solutions of capsaicin have been used to determine cough thresholds in subjects [1], and to test the effectiveness of antitussive agents [2]. However, several studies have demonstrated varying results of capsaicin tussigenic challenges [3,4]. We demonstrated in a prior study [5] the incomplete solubility of capsaicin in ethanol/saline solution, and the instability of stored capsaicin solutions. It is possible that these findings are potential sources of the variable results. We also showed that solutions less than 4 μM decreased in concentration after 2 months when stored in the dark at 4°C, while all concentrations stored at room temperature, whether protected from light or not, decreased in concentration after 4 months of storage.

While Tween-80 has been utilized by others [6-9] when making solutions of capsaicin in an empiric attempt to increase the solubility of capsaicin, these authors did not measure the actual concentrations of the solutions. Tween-80 (Sorbitan mono-9-octadecenoate poly(oxy-1.2-ethanediyl), also known as polysorbate 80, is an agent that is used as an emulsifier and dispersing agent for medications designed for internal use. It is very soluble in water, alcohol, and organic solvents such as toluene. Consequently, we sought to prospectively and objectively determine, for the first time, if the use of Tween-80 in solutions of capsaicin improves the actual concentration of freshly prepared solutions of capsaicin, and to determine if Tween-80 could result in maintaining the concentrations of stored solutions kept under various environmental conditions. Because capsaicin tussigenic challenges are being used more frequently to assess the sensitivity of the cough reflex and the efficacy of antitussive agents [10], it is clinically important to standardize all aspects of the capsaicin tussigenic challenge methodology.

## Methods

Two stock solutions of 128 μM concentrations of capsaicin were made. Solution 1 (without Tween-80) was made by dissolving 39 mg of capsaicin powder (Sigma Chemical Co., St Louis, MO) in 12.5 mL of 100% ethanol; this was then diluted with 0.9% sodium chloride solution to a total volume of 1 liter. Solution 2 (with Tween-80) was made by dissolving 39 mg of capsaicin powder in 5 mL of 100% ethanol. To this, 5 mL of Tween-80 (Fisher Scientific, Leicestershire, UK) was then added, and the solution was diluted with 0.9% sodium chloride solution to a total volume of 1 liter.

The two stock solutions of 128 μM concentration of capsaicin were repeatedly diluted 1:1 with 0.9% sodium chloride solution to make 2 sets of solutions of 64, 32, 16, 8, 4, 2, 1, and 0.5 μM. One set contained Tween-80 (from stock Solution 2) and the other set did not contain Tween-80 (from stock Solution 1). Aliquots of these freshly made solutions were then analyzed for actual concentration.

Additional aliquots of each of the freshly prepared solutions, both with Tween-80 and without Tween-80, were separated for storage under several environmental conditions. Samples of all nine of the concentrations of both the Tween-80 and non-Tween-80 solutions were stored at 4°C and protected from light. Samples of solutions of 4 μM, 8 μM, and 16 μM capsaicin concentrations were stored at room temperature and protected from light, and room temperature exposed to light. Finally, samples of solutions of 32 μM, 64 μM, and 128 μM were stored in a freezer at -20°C. Samples to be kept in the dark (at room temperature, refrigerated at 4°C, and frozen at -20°C) were placed in amber vials, and the vials were then stored in light protecting boxes. Samples to be stored exposed to light were placed in clear vials and stored on a laboratory shelf. Samples were tested at baseline and then every 2 months for a total of 4 months. During testing, each sample was tested twice. The concentration of each individual sample was determined by calculating the average of the 2 tests. However, if the results of the two tests on any one solution differed by more than 3%, they were discarded and two additional tests were performed to obtain data within the acceptable variations.

The concentrations of the solutions of capsaicin were measured by high performance liquid chromatography (HPLC). Measurements were made both via electrochemical detection and ultraviolet detection. Initial calibration tests demonstrated that electrochemical detection produced non-linear results for concentrations above 16 μM. Therefore, electrochemical detection was used only on the solutions of 16 μM or less, and ultraviolet detection was used for the 32 μM, 64 μM, and 128 μM concentrations. The HPLC system consisted of an ESA 540 autosampler (ESA, Inc., Chelmsford, MA), an ESA 580 pump, and a Spectra-Physics SP8490 UV detector (ThermoFinnigan, Waltham, MA) set at 280 nm. Data were acquired and analyzed by PC/Chrom+ software (H&A Scientific, Greenville, NC). The column was a Phenomenex Luna (C_18_, 150 × 4.6 mm, 5 μm particles, 100 Å pores) (Torrance, CA). The method was isocratic, with a flow rate of 1.5 mL/min and a run time of 12 minutes. Injection volume was 20 μL. Capsaicin eluted at 5 minutes.

The HPLC mobile phase consisted of 3 g of NaH_2_PO_4 _dissolved in 480 mL of purified H_2_O, to which 520 mL acetonitrile was added. This solution was adjusted to pH 4 with H_3_PO_4 _and filtered through a 0.2 μm nylon membrane prior to use. The mobile phase was not re-circulated because it was found that the introduction of Tween-80 significantly increased the retention time of capsaicin after repeated injections.

For statistical analysis, solutions stored at 4°C and protected from light were divided into "low concentration solutions" which included the 0.5, 1.0, and 2.0 μM concentrations, and "high concentration solutions" which included the 4.0, 8.0, 16.0, 32.0, 64.0, and 128.0 μM concentrations. Statistical analysis of the data was performed using paired t-tests and one-way

ANOVA. Calculations were performed using SSPS-12 software (SSPS Inc., Chicago, IL). The 0.05 level of significance was used throughout.

## Results

### Appearance of freshly prepared solutions

The initial stock solution containing Tween-80 was clear in color, while the non-Tween containing stock solution had a whitish, cloudy appearance. The subsequent concentrations of the non-Tween containing solutions became less cloudy with further dilution with saline as described in the Methods Section. The Tween-containing solutions remained clear even after further dilution.

### Results of baseline freshly prepared solutions

Predicted and actual initial concentrations of Tween-80 and non-Tween-80 containing solutions are summarized in Table 1. For each of the paired concentrations, those containing Tween-80 were consistently higher. The 128 μM stock solution of capsaicin prepared with Tween-80 had an actual baseline concentration of 120.5 μM, while the 128 μM stock solution prepared without Tween-80 had an actual baseline concentration of 92.7 μM. The average baseline concentration of all the freshly prepared solutions of capsaicin prepared with Tween-80 was 83.9 ± 6.8% of predicted, with a range of 74.4–94.1%. The average baseline concentration of the freshly prepared solutions without Tween-80 was 63.4 ± 3.4% with a range of 61.5–72.4%. The actual concentrations of all the Tween-80 containing solutions were significantly greater than the non-Tween-80 solutions (p < .001).

**Table 1:** Predicted and Actual Initial Concentrations of Tween-80 vs. non-Tween-80 Containing Solutions.

Actual Concentrations (% predicted)		
Predicted Concentration (in μM)	Tween-80	non-Tween-80

0.5	0.44 (88.4)	0.33 (67.4)
1.0	0.78 (78.1)	0.64 (63.5)
2.0	1.49 (74.4)	1.24 (62.2)
4.0	3.12 (78.0)	2.48 (62.0)
8.0	6.27 (78.4)	4.92 (61.5)
16.0	13.74 (85.9)	10.37 (63.1)
32.0	28.54 (89.2)	21.82 (68.2)
64.0	56.90 (88.9)	42.56 (66.5)
128.0	120.45 (94.1)	92.67 (72.4)

### Results of stored solutions containing Tween-80

#### Solutions stored at 4°C and protected from light

These data are summarized in Table 2. For individual concentrations of Tween-80 containing solutions stored at 4°C and protected from light, there was a significant decrease in the lower concentrations (0.5, 1, and 2 μM) compared to the higher concentrations (4, 8, 16, 32, 64, and 128 μM) after 2 months (p = .041). The low concentration solutions averaged a 20.1% decrease in concentration after 4 months while the high concentration solutions had a slight increase in average concentration of 1.2% after 4 months. The largest decrease in concentration was seen in the 0.5 μM solutions.

**Table 2:** Actual Concentrations of Tween-80 and non-Tween-80 containing solutions at 0, 2, and 4 months stored at 4°C and protected from light.

	Actual Concentration (% predicted)					
	Tween-80			Non-Tween-80		
Predicted Concentration (in μM)	0 month	2 months	4 months	0 month	2 months	4 months

0.5	0.44 (88.4)	0.23 (45.4)	0.24 (47.8)	0.33 (67.4)	0.24 (47.8)	0.27 (53.5)
1.0	0.78 (78.1)	0.59 (58.9)	0.59 (58.6)	0.64 (63.5)	0.55 (54.5)	0.58 (58.2)
2.0	1.49 (74.4)	1.30 (64.9)	1.47 (73.4)	1.24 (62.2)	1.06 (53.0)	1.18 (59.2)
4.0	3.12 (78.0)	2.97 (74.3)	3.32 (83.0)	2.48 (62.0)	2.36 (59.1)	2.49 (62.2)
8.0	6.27 (78.4)	5.94 (74.3)	7.10 (88.8)	4.92 (61.5)	4.37 (54.6)	4.74 (59.3)
16.0	13.74 (85.9)	11.76 (73.5)	12.62 (78.9)	10.37 (63.1)	8.32 (52.0)	8.99 (56.2)
32.0	28.54 (89.2)	26.50 (82.8)	27.30 (85.3)	21.82 (68.2)	21.42 (66.9)	21.37 (66.8)
64.0	56.90 (88.9)	56.19 (87.8)	55.87 (87.3)	42.56 (66.5)	40.32 (63.1)	42.37 (66.2)
128.0	120.45 (94.1)	115.07 (89.9)	119.68 (93.5)	92.67 (72.4)	43.26 (33.8)	52.61 (41.1)

#### Solutions stored at room temperature and protected from light

These data are summarized in Table 3. All the Tween-80 solutions stored at room temperature and protected from light had a significant drop in concentration after 2 months (p < .001) and 4 months (p < .001) compared to baseline concentrations.

**Table 3:** Actual Concentrations of Tween-80 and non-Tween-80 containing solutions at 0, 2, and 4 months stored at room temperature and protected from light, and room temperature exposed to light.

		Actual Concentration (% predicted)					
		Tween-80			Non-Tween-80		
Predicted Concentrations (in μM)	Environment	0 month	2 months	4 months	0 month	2 months	4 months

4.0	RT/dark	3.12 (78.0)	1.24 (31.1)	0.79 (19.8)	2.48 (62.0)	0.45 (11.2)	0.04 (1.1)
8.0	RT/dark	6.27 (78.4)	3.93 (49.1)	3.47 (43.4)	4.92 (61.5)	1.06 (13.3)	0.61 (7.6)
16.0	RT/dark	13.74 (85.9)	8.93 (55.8)	8.46 (52.9)	10.37 (63.1)	3.62 (22.6)	1.47 (9.2)
4.0	RT/light	3.12 (78.0)	0.13 (3.2)	0.03 (0.7)	2.48 (62.0)	0.08 (2.0)	0.00 (0.0)
8.0	RT/light	6.27 (78.4)	0.74 (9.2)	0.70 (8.7)	4.92 (61.5)	0.30 (3.7)	0.00 (0.0)
16.0	RT/light	13.74 (82.7)	3.44 (21.5)	1.73 (10.8)	10.37 (63.1)	2.70 (16.9)	1.12 (7.0)

RT/dark = room temperature and protected from light, RT/light = room temperature and exposed to light

#### Solutions stored at room temperature and exposed to light

These data are summarized in Table 3. Similarly, all Tween-80 containing solutions stored at room temperature and exposed to light had a significant drop in concentration at 2 months (p = .032) and 4 months (p = .042).

#### Solutions stored at -20°C

These data are summarized in Table 4. All the Tween-80 containing solutions had a significant decrease in concentration after 4 months when stored at -20°C and protected from light (p < .001) when compared to baseline concentrations.

**Table 4:** Actual concentrations of Tween-80 and non-Tween-80 containing solutions at 0 and 4 months stored at -20°.

	Actual Concentration (% predicted)			
	Tween-80		Non-Tween-80	
Predicted Concentration (in μM)	0 Month	4 Months	0 Month	4 Months

32.0	28.54 (89.2)	22.62 (70.7)	21.82 (68.2)	15.87 (49.6)
64.0	56.90 (88.9)	47.81 (74.7)	42.56 (66.5)	30.53 (47.7)
128.0	120.45 (94.1)	104.32 (81.5)	92.67 (72.4)	69.63 (54.4)

### Results of stored solutions without Tween-80

#### Solutions stored at 4°C and protected from light

These data are summarized in Table 2. For solutions stored at 4°C and protected from light, there were significant decreases in the low concentration solutions compared to the high concentration solutions after 2 months (p = .012). After 4 months, the low concentration solutions averaged a 7.4% decrease in concentration, while the high concentration solutions decreased an average of 7.6%. The largest decreases were seen in the 0.5 μM (13.9% decrease) and 128 μM (31.3% decrease) solutions.

#### Solutions stored at room temperature and protected from light

These data are summarized in Table 3. All the non-Tween-80 solutions stored at room temperature and protected from light had a significant drop in concentration after 2 months (p < .001) and 4 months (p < .001) compared to baseline concentrations.

#### Solutions stored at room temperature and exposed to light

These data are summarized in Table 3. Significant decreases in concentration were also seen in the solutions without Tween-80 that were stored at room temperature and exposed to light at 2 months (p = .002) and 4 months (p = .013) compared to baseline.

#### Solutions stored -20°C

These data are summarized in Table 4. All the solutions without Tween-80 stored at -20°C and protected from light had significant decreases in concentration at 4 months compared to baseline (p < .001).

### Comparative results of Tween-80 vs. non-Tween-80 stored solutions

#### Solutions stored at 4°C and protected from light

For the solutions stored at 4°C and protected from light, there was a significant drop in the concentration of the non-Tween-80 containing solutions when compared to the Tween-80 solutions at 2 and 4 months (p = .003) (Table 2).

When comparing the lower concentrations (0.5 μM and 1 μM) of solutions with and without Tween-80 in Table 2, the trend of the data suggest that the non-Tween-80 containing solutions of these 2 concentrations might be more stable after 4 months of storage than the Tween-80 containing solutions. However, this trend was not found to be significant (p = .189).

#### Solutions stored at room temperature and protected from light

While there were significant decreases in all solutions stored at room temperature and protected from light, the decrease in the non-Tween-80 containing solutions was significantly greater than the Tween-80 containing solutions at 2 and 4 months (p < .001) (Table 3).

#### Solutions stored at room temperature and exposed to light

Identical results were seen with all solutions stored at room temperature and exposed to light. Whether containing Tween-80 or not, there were significant decreases in all concentrations at 2 months (p = .001) and 4 months (p < .001) when compared to the baseline concentration (Table 3). The decreases in concentrations of the non-Tween-80 containing solutions were greater at 2 months (p < .001) and 4 months (p < .001) than the decreases in the Tween-80 containing solutions.

While all concentrations of solutions stored at room temperature significantly decreased at 2 and 4 months compared to baseline, the decreases were greater in the solutions exposed to light vs those protected from light (p < .001 at 2 and 4 months).

#### Solutions stored at -20°C

When compared to the baseline concentration, both the Tween-80 containing solutions and those without Tween-80 had a significant decrease in concentration after 4 months when stored at -20°C and protected from light (p < .001). There was no significant difference between the decrease in concentration of the Tween-80 compared to the decrease in the non-Tween-80 containing solutions after 4 months (p = .136).

## Discussion

From our study, two notable findings emerged that have not been previously reported regarding the use of Tween-80 in preparing capsaicin solutions. First, our results demonstrate that the use of Tween-80, an emulsifier and dispersing agent, significantly improved the actual concentration of freshly prepared solutions of capsaicin compared to solutions not containing Tween-80. Second, the use of Tween-80 did not prevent solutions from decreasing in concentration when stored at room temperature and protected from light, room temperature and exposed to light, or frozen at -20°C after 4 months. This trend is consistent with the results of our first study in which we analyzed only solutions made without Tween-80 [5].

With respect to the use of Tween-80 to prevent the breakdown of capsaicin solutions over time, when stored at various environmental conditions, we found that solutions of 2 μM and less of both the Tween-80 containing and non-Tween-80 containing solutions did significantly decrease in concentration after 2 months compared to the higher concentration. However, it did appear that the higher concentration of Tween-80 containing solutions were more stable after 4 months when stored at 4°C and protected from light compared to the non-Tween-80 solutions. The non-Tween-80 data in this study are similar to the results in our initial study of non-Tween-80 containing solutions [5]. Although the actual, initial concentrations of the freshly prepared non-Tween-80 solutions were lower in this study compared to our first [5], we attribute the difference to our using a newer assay in this study. After testing both assays in the laboratory, we found that the assay used in this current study provided more consistent results. Despite the differences in the values between our two studies, the overall results with regard to incomplete solubility and the breakdown of the stored solutions were similar. Because the serial dilutions of the freshly prepared solutions of capsaicin consistently resulted in concentrations nearly half of the previous solution's concentrations (Table 1) and this finding appeared to be independent of whether or not Tween-80 was used in the initial stock solution, it appears that the major benefit of using Tween-80 when preparing the solutions appears to be the increase in concentration of the initial stock solution.

Tween-80 is commonly used in pharmaceutical products to improve solubility of water insoluble medications. While Tween-80 is felt to be safe for intravenous and inhaled use, there have been a few reported cases of toxicity and hypersensitivity that have been associated with Tween-80 found in some medication preparations. Liver and kidney damage in infants who received intravenous vitamin E has been attributed to Tween-80 [11], as has severe acute hepatitis seen in patients receiving intravenous amiodarone [12]. While we are not aware of any reported toxicities associated with the inhaled use of Tween-80, there is a case report of contact dermatitis that was felt to have developed as a result of repeated exposure to Tween-80 found in budesonide solution [13]. Nevertheless, a retrospective analysis of 122 studies containing a total of 4833 subjects provides some evidence that capsaicin cough challenges in humans are safe [14], but it is unclear how many of these studies used Tween-80 in the preparation of the capsaicin solutions and how good data capture was.

### Methodological considerations

While there have been several reported methods for making capsaicin solutions without Tween-80 [1,2,5], we are aware of only one other published reported method using Tween-80 [6-9]. In all 4 of these studies by Fujimura et al, the identical method was used to prepare the capsaicin solutions with Tween-80, but capsaicin solubility was not objectively assessed. Our method for making solutions of capsaicin with Tween-80 differed slightly from the method used by Fujimura et al. Specifically, Fujimura et al dissolved 30.5 mg of capsaicin powder in 1 ml of Tween-80 and 1 ml of ethanol, and then diluted the mixture with 8 ml of 0.9% saline. We dissolved 39 mg of capsaicin powder in 5 ml of Tween-80 and 5 ml of ethanol, and then diluted the mixture with 0.9% saline to a total volume of 1 liter of 128 μM concentration. We chose to use higher amounts of both ethanol and Tween-80 in an attempt to maximize the solubility of the capsaicin powder. It is unclear if this slight difference in methods between our study and the method proposed by Fujimura et al would result in significantly different concentrations, or significantly impact the decay of the solutions stored at various environmental conditions. To our knowledge, only our previous study [5] and this current study evaluated the actual solubility of capsaicin and the decay of capsaicin concentrations over time under various environmental conditions.

### Clinical Implications for preparing and storing solutions of capsaicin

While the addition of Tween-80 in preparing fresh solutions of capsaicin improves the actual concentration of capsaicin with our method of preparation, Tween-80 does not facilitate complete solubility of capsaicin. Also, the addition of Tween-80 does not prevent the breakdown of capsaicin solutions over time. The FDA considers Tween-80 to be safe for use in intravenous and inhaled medications, but we know of no prospective safety studies that demonstrate that there is no increased risk of side effects associated with inhaled capsaicin solutions made with Tween-80. Therefore, we recommend using Tween-80 cautiously until prospective studies are available to confirm its safety.

When prepared capsaicin solutions are to be used, we recommend the following: When preparing fresh solutions of capsaicin, Tween-80 should be added because it improves the actual concentration. Nevertheless, even with use of this this emulsifier and dispersing agent, it should be appreciated that the actual concentration will still be less than 100%.

If solutions of capsaicin are to be stored for future use, we recommend making a stock solution of 128 μM with Tween-80, and storing the solution at 4°C and protected from light, as solutions of 128 μM made without Tween significantly breakdown when stored at these conditions after 4 months (see table 2) . Solutions should be discarded after 4 months.

When making solutions to be stored, the addition of Tween-80 does not appear to prevent a decrease in the 0.5 μM, 1.0 μM, and 2.0 μM concentrations after 2 months when stored at 4°C and protected from light. Tween-80 also does not prevent the decrease in concentration of solutions stored at room temperature or frozen at -20°C.

## Conclusion

While the inherent difficulty of forcing capsaicin into solution cannot be eliminated, it can be improved with Tween-80. To decrease the variability of results of capsaicin tussigenic challenges, it is important to standardize all aspects of the methodology including preparing and storing capsaicin solutions. Until further refinements in capsaicin solution preparation are forthcoming that improve upon the present situation, we recommend preparing and storing capsaicin solutions according to the methods and results of this study.

## Competing interests

The authors declare that they have no competing interests.

## Authors' contributions

SK, RI, and RD conceived the study and participated in the design of the study, SK and RI drafted the manuscript, SK performed the statistical analysis, RD, MB, and TM prepared the solutions, oversaw the storage of the solutions, and performed the analysis of all the solutions. All authors read and approved the final manuscript.
